# Retroperitoneal Abscess Formation as a Result of Spilled Gallstones during Laparoscopic Cholecystectomy: An Unusual Case Report

**DOI:** 10.1155/2012/573092

**Published:** 2012-11-26

**Authors:** Grigoris Chatzimavroudis, Stefanos Atmatzidis, Basilis Papaziogas, Ioannis Galanis, Ioannis Koutelidakis, Triantafyllos Doulias, Petros Christopoulos, George Papadakis, Konstantinos Atmatzidis, John Makris

**Affiliations:** 2nd Surgical Department, School of Medicine, “G. Gennimatas” General Hospital, Aristotle University of Thessaloniki, 54635 Thessaloniki, Greece

## Abstract

One of the complications of laparoscopic cholecystectomy for gallstone disease that seems to exceed that of the traditional open method is the gallbladder perforation and gallstone spillage. Its incidence can occur in up to 40% of patients, and in most cases its course is uneventful. However in few cases an abdominal abscess can develop, which may lead to significant morbidity. Rarely an abscess formation due to spilled and lost gallstones may occur in the retroperitoneal space. We herein report the case of a female patient who presented with clinical symptoms of sepsis six months following laparoscopic cholecystectomy. Imaging investigations revealed the presence of a retroperitoneal abscess due to retained gallstones. Due to patient's decision to refuse abscess's surgical drainage, she underwent CT-guided drainage. The 24-month followup of the patient has been uneventful, and the patient remains in good general condition.

## 1. Introduction

Laparoscopic cholecystectomy (LC) has been established as the gold standard method for the treatment of gallstone disease due to its advantages, including less postoperative pain, shortened hospitalization, faster recovery, and improved cosmesis. However, LC is not without complications; in fact, specific complications can occur with higher frequency in LC than in traditional open approach, and gallbladder perforation with subsequent bile and gallstone spillage is included among them. Though common in incidence, ranging from 8% to 40% [1], the clinical importance of gallbladder perforation and gallstone spillage still remains unclear. Abscess formation due to lost gallstones occurs rarely and predominantly develops intraperitoneally [2]. We report the case of a patient with retroperitoneal abscess formation six months following LC.

## 2. Case History

A 72-year-old woman underwent LC due to symptomatic gallstone disease. Her past medical history was uneventful. Intraoperatively, the gallbladder was perforated resulting in bile and gallstone spillage into the peritoneal cavity. The abdominal cavity was irrigated with 1 lt of normal saline to dilute and aspirate bile and gallstones; however the retrieval of the spilled gallstones was incomplete as a combined result of their large number and small size. The postoperative course of the patient was uneventful. 

Six months postoperatively, the patient presented to our department complaining of high fever (body temperature up to 39.2°C), chills, and constant pain in the right lumbar region for two days. On clinical examination the right hypochondrium and right lumbar region were both tender. Blood tests revealed white blood cell count 13.200/mL, neutrophils 87.3%, and C-reactive protein 14.9 mg/dL (normal value <0.5 mg/dL). 

**Figure 1:**
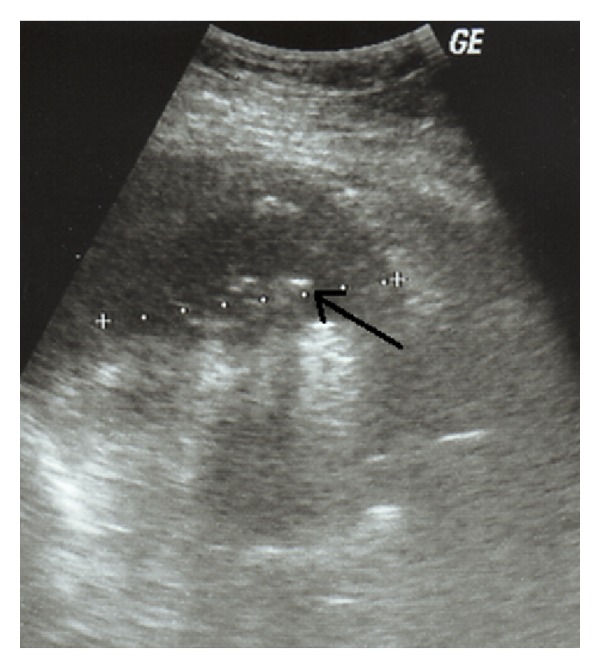
Ultrasound examination showing the presence of a retroperitoneal abscess with hyperechoic foci (spilled gallstones) (arrow).

**Figure 2:**
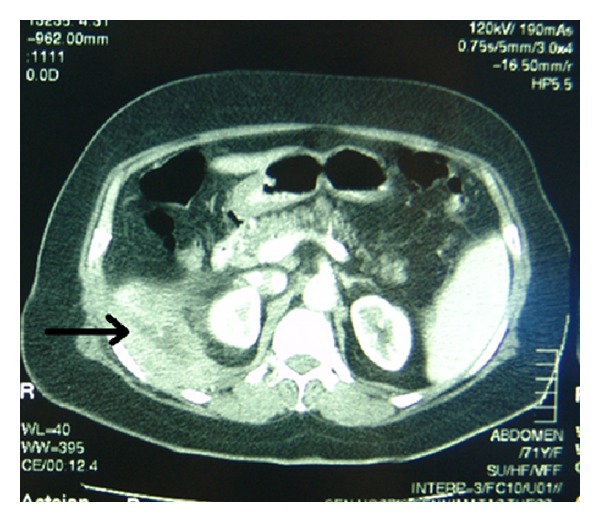
CT scan showing a retroperitoneal abscess (arrow) due to retained gallstones.

The patient underwent ultrasound examination which revealed an abscess cavity in the right retroperitoneal space, containing multiple hyperechoic foci (Figure 1). Due to the recent history of LC complicated with spilled gallstones, the diagnosis of a retroperitoneal abscess was suspected, which was confirmed by the computed tomography (CT) scan findings (Figure 2). 

The patient was recommended to undergo surgical drainage of the abscess, but she refused. Alternatively, she underwent percutaneous CT-guided drainage of the abscess. The content of the abscess cavity was sent for microbiological analysis which grew *Klebsiella pneumoniae*. The patient was given intravenous antibiotics (ciprofloxacin 400 mg ×2 and metronidazole 500 mg ×3) for seven days, and afterwards she was discharged from hospital in good condition, entering an intensive follow-up program. Twenty-four months after abscess drainage, the patient has remained asymptomatic.

## 3. Discussion

Based on the statistics, a complication that almost every laparoscopic surgeon will face at least one time in his surgical career is gallbladder perforation with subsequent bile and gallstone spillage. The fact that laparoscopic cholecystectomy is one of the most common surgical operations, combined by the high incidence (up to 40%) of gallbladder perforation [1, 3], undoubtedly justifies the above statement. Fortunately in the vast majority of cases with spilled gallstones, the postoperative course of patients is unremarkable. Reviewing six studies covering 18280 LCs, Woodfield et al. estimated the risk of complications due to split gallstones to be 1,7 cases per 1000 LCs [2]. Similarly, in a more recent article representing a single center experience, Tummer et al. reported that only seven out of 1528 (0.45%) patients with LC presented with complications due to retained gallstones [4].

Zehetner et al. reviewed all the reported complications from lost gallstones and found that abscesses in the abdominal wall and intra-abdominal abscesses were the most frequently reported complications [5]. In 2002 Papasavas et al. reviewed 127 cases of spilled gallstones presenting with various clinical manifestations. Of these cases, more than 60% presented with abdominal abscess formation (intraperitoneal plus abdominal wall abscesses), while 10% suffered from retroperitoneal abscess [6]. Since 2002 only five new cases of retroperitoneal abscess due to retained gallstones have been reported [7–11].

Other than abscess formation complications due to spilled gallstones include fistula formation, intestinal obstruction, pleural empyema, and broncholithiasis [5].

It remains unclear and a matter of controversy whether gallbladder perforation with bile and stone spillage during LC should be an indication for conversion to laparotomy. According to Brockmann et al., risk factors for complications after gallstone spillage are old age, stone size >15 mm, number of spilled stones >15, pigment stones, and infected bile [1]. However even for these cases the majority of authors do not advice conversion to open surgery [2, 4, 5, 11]. Instead, it is widely recommended to remove as many of the spilled stones as possible by laparoscopic means (e.g., graspers, 10 mm suction device) and intensively irrigate the peritoneal cavity. Moreover we believe that it is of highest importance to clearly document the incidence of gallstone spillage in the medical report and inform the patient in order to early recognize signs of any possible complication.

It is almost universally accepted that treatment of an abscess due to lost gallstones should include not only abscess drainage and antibiotic administration but also removal of the lost gallstones, otherwise the abscess might recur. Though we strongly agree with this approach, our patient's treatment did not include gallstone removal because she denied any surgical intervention. Two years after percutaneous drainage of the abscess, she has remained asymptomatic but still on close followup.

In conclusion, our reported case shows that, though rarely, spilled gallstones during LC can lead to severe morbidity and emphasizes the importance of having high index of suspicion for complications in such patients in order to early recognize and treat them appropriately.
